# Revisiting the Timing of Insemination at Spontaneous Estrus in Dairy Cattle

**DOI:** 10.3390/ani12243565

**Published:** 2022-12-16

**Authors:** Fernando López-Gatius

**Affiliations:** 1 Agrotecnio Centre, 25198 Lleida, Spain; lopezgatiusf@gmail.com; 2 Subunit, Transfer in Bovine Reproduction SLu, 22300 Barbastro, Spain

**Keywords:** cervix, estrus detection aids, gamete ageing, ovary

## Abstract

**Simple Summary:**

The possibility of artificial insemination (AI), mainly in dairy cattle, revolutionized animal and human reproduction procedures over the 20th century. In effect, AI in dairy cattle is considered the most important technological advancement in the field of animal breeding. Cows show estrous behavior over an 8-to-20 h period yet they are refractory to the bull some 10–12 h before ovulation. As spermatozoa need to persist in the female reproductive tract for 6–8 h before they are capable of fertilization, in the past 80 years the time interval for AI in cattle with fresh or frozen-thawed semen has been 24–6 h before ovulation. Recent evidence suggests this interval should be reduced.

**Abstract:**

Cows show spontaneous estrus over 8–20 h but become refractory to the bull about 10–12 h before ovulation. This indicates that ovulation occurs 10–12 h after the end of estrus behavior, yet spermatozoa from the bull ejaculate need to undergo maturation and capacitation for 6 to 8 h in the female reproductive tract before they are capable of fertilization. Traditionally, the onset of estrus has been considered the best timing for artificial insemination (AI) in cattle, that is, 6 to 24 h from the first signs of estrus. However, recent findings suggest this interval should be reduced to 16 to 6 h before ovulation, bringing it closer to the end of estrus. In this review, the end of estrus rather than its onset is proposed as the best guide for AI timing in dairy cattle, and physiological indicators of late estrus are discussed such as relaxation of the intravaginal part of the uterus, a lower cervical mucus viscosity and a softer pre-ovulatory follicular consistency as simple cues indicating a cow is ready for service.

## 1. Introduction

Cattle, like other ruminants, have been used to transform vegetable products into milk and meat from the beginning of agriculture, cattle breeding being one of the pillars of economic development since the Neolithic period [1]. In fact, social relationships among animal species, man being one of them, have been key to the evolution of the human species [2]. Examples of the outcome of these relationships include human fertility concepts derived from early knowledge of animal physiology [3]; man–cow interactions [4] leading to the genetic mutation that enables humans to produce lactase so that milk can be ingested over a lifetime; or the production of a vaccine against smallpox using material from cows since the 18th century [5]. More recently, the introduction of artificial insemination (AI), mainly in dairy cattle, revolutionized procedures of animal and human reproduction over the 20th century [6,7,8]. Indeed, for more than 80 years now, AI in dairy cattle has been considered the most important technological advancement in animal breeding [9,10,11]. The development of AI was prompted by the intensification of dairy farming. The reproductive records of cows are essential for AI. Additionally, the training of inseminators is a significant contribution to the successful commercial application of AI to dairy cattle breeding [12,13]. In consequence, inseminator training and improvements in the act of inseminating remain topics of great interest [14,15,16,17]. However, the correct detection of estrus remains a challenge [18,19,20,21,22] determining that some 20% of inseminations may be performed in pregnant cows [21].

Behavioral signs of estrus are a predictor of the time of ovulation and a guide to the optimal time of insemination. It is generally accepted that ovulation occurs 10–12 h after the end of estrus. Cows may show a period of standing estrus of 8–20 h but become refractory to the bull about 10–12 h before ovulation. Spermatozoa need to remain in the female reproductive tract for 6 to 8 h before they are capable of fertilization, and capacitated spermatozoa must be present in the fallopian tubes at the moment of ovulation [23,24,25,26,27]. The risk of the ageing of gametes, particularly the postovulatory oocyte, becomes critical at the time of AI in cattle [28,29,30]. Owing to the challenges of correctly detecting estrus, hormonal protocols for fixed-time AI (FTAI) have been incorporated into routine intensive dairy farming reproductive management programs [31,32,33,34]. However, most Ais worldwide are performed at spontaneous estrus and hormone treatments have met with strong consumer opposition in some countries. In addition, the use of sex-sorted semen has increased enormously over the last decade [35,36,37,38] and, in beef cattle, this practice has recently been recommended only in cows expressing estrus following FTAI protocols [39,40]. In the present report, findings are reviewed that could help identify the optimal time of AI in dairy cattle undergoing spontaneous estrus.

## 2. The Timing of Insemination

The optimal time of AI was established using fresh semen in the classic studies by Trimberger and Davis [41] and Trimberger [42]. Cows were examined by palpation per rectum at two-hour intervals during and following the standing behavior of estrus (not secondary signs) to locate the end of estrus and time of ovulation [42]. Highest conception rates (79%) were obtained when cows were inseminated in the middle to end of estrus (24 to 6 h before ovulation). For comparative purposes, these early data provided by Trimberger [42] are presented for 12 h intervals in Table 1.

These findings led to the extensively used “a.m.-p.m.” guidelines. These state that cows detected to be in estrus in the morning (a.m.) should be submitted for AI that afternoon (p.m.), and cows in estrus in the afternoon should be inseminated the next morning. Subsequent studies confirmed earlier work in which best results were obtained when heifers or cows were inseminated with fresh or frozen semen 7 to 18 h after the detection of estrus [43,44,45,46,47,48]. However, in two extensive studies involving a total of 51,947 Ais, differences were not detected between cows receiving a single mid-morning AI, when most cows would be in early or mid-estrus [49], and cows inseminated once daily compared to the a.m.–p.m. guidelines [50]. These wide ranges in the optimum time to inseminate cows may reflect the effect of a variable frequency of estrus observation, or variable efficiency of the technologies used to detect cows in estrus.

More recently, using frozen-thawed semen in tie-stalled cows, Sumiyoshi et al. [22] obtained a conception rate of 63% when AI was performed 30 to 6 h before ovulation, which was significantly higher than the conception rates resulting from inseminations performed earlier or later than this interval. Estrus was confirmed according to eight estrous signs: first, swelling and hyperemia of the vulva by visual observation, then swelling and relaxation of the intravaginal part of the uterus and opening of the external uterine orifice using a sterile speculum, viscosity of the cervical mucus, and contraction of the uterus and diameter of the uterine horn on transrectal ultrasonography. Ovulation was assessed at 6 h intervals by rectal ultrasonography. Table 1 shows the data recorded at 12 h intervals. 

The use of automated estrus detection technologies potentially offers a high level of accuracy [51,52,53,54,55]. Using frozen-thawed semen, Roelofs et al. [56] investigated the effects of the insemination-ovulation interval on embryo quality seven days post-AI. The time of ovulation following estrus onset (as determined through pedometers or visual observation) was assessed every 4 h by rectal ultrasonography. When data were stratified by 12 h intervals, good quality embryo rates were significantly higher for AI before ovulation than after ovulation (Table 1). Accordingly, the highest percentage of good quality embryos (67.6%) was detected when AI was performed 24 to 12 h before ovulation. Within this interval, the percentage of good embryos increased to 89% when AI was performed 16 to 13 h before ovulation. In the same study, fertilization rates were significantly higher when AI was conducted between 36–24 h and 24–12 h before ovulation (85% and 82%, respectively) compared to AI after ovulation (56%) [56].

In a further study using frozen-thawed semen, the highest pregnancy rate (50.8%) was recorded for AIs performed 16 to 0 h before ovulation [57]. Pregnancy rates were significantly lower for AIs executed 32 to 16 h before ovulation (28.7%) and for AIs executed after ovulation (20%). The time of ovulation was assessed every 12 h by rectal ultrasonography following AI [57]. Using frozen sex-sorted semen, which is a sub fertile inseminate compared to conventional semen [35,36,37,38], AI intervals 0 to 32 h from the onset of estrus did not affect conception rates [58]. However, conception rates were higher in response to AI conducted −4 to 4 h (57.1%) compared to −12 to −4 h (37.7%) or 12–20 h (30.3%) from the end of estrus, whereas using conventional semen, the AI interval from estrus onset and end did not affect conception rates. These authors suggested that the time of estrus end is a better indicator of optimal AI timing for sex-sorted semen than the time of estrus onset. 

While high conception rates can be obtained when AI is performed within a wide range of hours, 30 to 6 h before ovulation, it seems that embryo quality and ovulation time [56,57,58] suggest this interval should be reduced. This reduction could improve pregnancy rates under certain circumstances such as when semen quality is poor.

## 3. Is End-Estrus the Best Guide for the Timing of Insemination?

Based on standing estrus as the most accurate sign of this stage, intervals between estrus onset and ovulation range from 16 to 61 h, averaging 27 to 40 h [59,60,61,62]. In contrast, intervals between estrus end and ovulation range from 3 to 18 h and average at 9 to 12 h [42,43,63]. Therefore, the end of standing behavior is a more accurate predictor of ovulation time than estrus onset. The problem, however, is that not all cycling cows show standing estrus [42,43,64,65,66,67]. In fact, the percentage of cows in estrus that stand to be mounted has declined from 80% to 50% over the last decades [68]. Grouping different behavioral estrous signs, 95% of cows were detected to be in estrus, intervals from estrus onset and end to ovulation being highly variable among cows [64]. High individual variability in both intervals relative to the time of ovulation has also been described using automated activity monitors [53,57,69,70]. This limits their use as a practical predictor of ovulation. However, both the visual and automated detection of estrus indicate there is an increase in physical activity that peaks and then declines. This makes it easy to conduct AI towards the end of each cow’s interval of estrus. Currently the use of automated systems does not usually exceed 20% of dairy herds [71,72] and standing estrus can be detected in at least 50% of cows on remaining farms [64].

## 4. Physiological Indicators of Late Estrus

**Figure 1:**
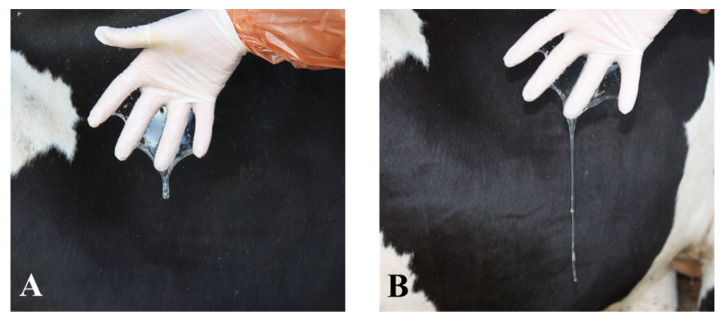
Cervical mucus viscosity can be used to differentiate between early estrus (mucus less hydrated) (**A**), and late estrus (mucus more hydrated) (**B**) [73,74,75,76]. Mucus samples were obtained from the cranial vagina with the help of a plastic inseminating sheath attached to a 50 mL syringe [75]. The time interval from A to B was 16 h.

Along with decreased estrous behavior activity, some physiological signs can be used as predictors of end estrus at the time of AI. Using a sterile speculum, relaxation of the intravaginal part of the uterus is very apparent from 18 to 6 h before ovulation [73,74] and this has been considered the optimal time for AI [74]. Between 24 and 6 h before ovulation, the cervical mucus reduces its viscosity to a maximum, becoming highly hydrated [73,75], its ultrastructural and physical properties markedly changing at the end of manifest estrus [76]. A lower cervical mucus viscosity at AI has been associated with a higher pregnancy rate [46]. To time AI, the routine testing of mucus obtained from the vagina [77] may help differentiate between early estrus, when mucus is less hydrated (Figure 1A), and late estrus, when it is more hydrated (Figure 1B). Finally, the consistency of the pre-ovulatory follicle at AI may also indicate the optimal time for AI. During estrus, rectal palpation of the pre-ovulatory follicle, irrespective of its size, reveals the sequence: young follicle (early estrus: firm/soft), mature follicle (late estrus: very soft), and finally ovulatory follicle (after estrus: emptied) [78,79,80]. In an extensive study involving 2365 AIs, the highest pregnancy rate was observed in cows with a mature follicle at AI [81]. In this study, the warm period of the year, which clearly impairs fertility in the geographic area of the study [82,83], had no negative impact on the fertility of cows with a mature follicle at insemination [81].

Careful examination of the reproductive organs should cause no harm to the animal [84]. Uterine stimulation triggers the release of oxytocin [85,86] and palpation of the ovaries may shorten the interval from end estrus to ovulation [63]. These effects are similar to those induced by mating [85,87] or by traditional AI techniques [85]. The incorporation of exams of the genital organs into AI routines should improve the success of this procedure.

## 5. Concluding Remarks

The onset of estrus behavior as a predictor of the time of ovulation has traditionally been considered the best guide to time AI. However, this reference has two negative aspects. First, its high individual variability and, second, it is difficult to accurately locate the true onset of estrus. In contrast, once a cow has been detected in estrus, its peaking activity and decline in this activity towards the end of estrus may be a better reference to estimate when ovulation will take place. 

The time interval 24 to 6 h before ovulation is widely accepted as the optimal time for insemination. However, recent findings suggest this interval should be reduced to 16 to 6 h before ovulation [56,57,58]. In other words, AI closer to end-estrus is a better insemination time. As ovulation occurs 10–12 h following the end of estrus behavior [23,24,25,26], physiological indicators of late estrus such as ready for service may be simply and directly detected at AI [73,74,75,81]. Thus, by better predicting the ovulation time, pregnancy rates to AI may be improved [57].

Insemination of the cow is the last, but not the least important stage of reproduction. The act of insemination requires knowledge of the reproductive anatomy and physiology of the cow, practice, and a good tactile-mental image of the normal estrus signs of the genital organs. The inseminator’s first objective should be to positively identify the optimal time of AI in a cow detected in estrus. Ovarian, uterine and vaginal findings may be significant predictors of AI outcomes. Rejection of cows that are not ready for service is a further opportunity to improve the success of AI programs. This type of intervention should increase the pregnancy rate during the optimum breeding period.

## Figures and Tables

**Table 1:** Effects of insemination-ovulation interval on conception and rate of good quality embryos.

AI-ovulation Interval ^(a)^	Conception (*n* = 132) [42]	Conception (*n* = 100) [22]	Good Quality Embryos (*n* = 122) [56]
Before ovulation			
>24 h	8/15 (53.3%)	10/26 (38.5%)	11/27 (40.7%) *
12–24 h	23/29 (79.3%)	18/28 (64.3%)	23/34 (67.6%) **
0–12 h	19/28 (67.9%)	16/31 (51.6%)	12/29 (41.4%) ***
After ovulation			
0–12 h	14/40 (35%)	3/12 (25%)	2/32 (6.3%) ****
Ovulation assessed	Every 2 h	Every 6 h	Every 4 h

**^(a)^** Values with different superscripts within columns denote significant differences detected by logistic regression procedures (*, ****: *p* = 0.01; **, ****: *p* < 0.0001; ***, ****: *p* = 0.004).

## Data Availability

All the data provided have been extracted from the cited references.
